# The Analogs of Temporin-GHa Exhibit a Broader Spectrum of Antimicrobial Activity and a Stronger Antibiofilm Potential against *Staphylococcus aureus*

**DOI:** 10.3390/molecules24224173

**Published:** 2019-11-18

**Authors:** Zhipeng Xie, Hanqi Wei, Jiahui Meng, Tong Cheng, Yanting Song, Manchuriga Wang, Yingxia Zhang

**Affiliations:** 1 Key Laboratory of Tropical Biological Resources of Ministry of Education, School of Life and Pharmaceutical Sciences, Hainan University, Haikou 570228, China; xiezhipeng1994@163.com (Z.X.); w370694686@163.com (H.W.); frank1026855189@gmail.com (J.M.); ct1498156623@163.com (T.C.); songyanting3323@hotmail.com (Y.S.); 2 College of Animal Science and Technology, Hainan University, Haikou 570228, China

**Keywords:** antimicrobial peptides, temporin-GHa, antibiofilm, peptide design, *Staphylococcus aureus*

## Abstract

The abuse of antibiotics has led to the emergence of multidrug-resistant bacteria, which is becoming a serious worldwide problem people have to face. In our previous study, temporin-GHa (GHa) cloned from *Hylarana guentheri* showed antimicrobial activity against Gram-positive bacteria. In order to improve its therapeutic potential, we used a template-based and a database-assisted design to obtain three derived peptides by replacing the histidine at both ends of GHa with lysine, which exhibited faster and stronger bactericidal activity and a broader spectrum than the parent peptide. GHaK and GHa4K targeted to the bacterial membrane to exert their antibacterial activities at a faster membrane damage rate. The derived peptides inhibited the initial adhesion and the formation of *Staphylococcus aureus* biofilms, and eradicated the mature biofilms, which indicated that the derived peptides effectively penetrated the biofilm and killed bacteria. The therapeutic index (TI) and cell selectivity index (CSI) of the derived peptides increased significantly, which means a broader therapeutic window of the derived peptides. The derived peptides with improved activity and cell selectivity have the potential to be the promising candidates for the treatment of *S. aureus* infections. Our research also provides new insights into the design and development of antimicrobial peptides.

## 1. Introduction

Infectious diseases have been one of the most life-threatening health problems worldwide throughout human history. The discovery of antibiotics lit up the dawn light to people who suffered from infections and saved their lives, making antibiotics the ultimate weapon against bacteria. However, due to the misuse of conventional antibiotics, bacteria have developed various strategies to avoid the killing efficacy of antibiotics [1]. In recent years, bacteria have accelerated their adaptation steps to resist antibiotics, developing multidrug-resistance to more antibiotic classes. Multidrug-resistant bacteria have become the severe therapeutic challenge in the clinic [2,3]. *Staphylococcus aureus* is a symbiotic bacterium of humans, distributed in the skin, nose, respiratory tract, and digestive system, which is the main pathogen causing skin and respiratory infections [4,5]. *S. aureus* infections can be treated with some antibiotics, including penicillin, oxacillin, or vancomycin, but the multidrug-resistant bacteria, like methicillin-resistant (MRSA) and vancomycin-resistant strains (VRSA), are very difficult to treat, leading to serious infections in hospital [6,7].

Biofilms are defined as a community of bacteria, where cells attach to the surface and excrete the extracellular substrates to provide the matrix to embed in [8]. Compared with single planktonic bacteria, the biofilms show stronger resistance to antibiotics and the host immune system, making infections chronic and repetitive, and leading to serious clinical problems [9]. To treat the biofilm infections appeals us to develop new antimicrobial agents that would inhibit the formation of biofilms or eliminate them. The promising candidates are antimicrobial peptides (AMPs), which are widely found in microorganisms, plants and animals, playing a major role in the innate immune system to protect the host against infections [10]. AMPs are short, positively-charged amphipathic peptides, which show the rapid and broad-spectrum antimicrobial activity against Gram-negative bacteria, Gram-positive bacteria, viruses, and fungi [11]. Most AMPs target the bacterial membrane to disrupt the membrane structure, and others interfere with intracellular metabolic processes, such as protein synthesis, and DNA duplication [12].

In our previous study, we cloned the temporin-GHa (GHa) peptide from the frog *Hylarana guentheri*, which exhibited the stronger antimicrobial activity against Gram-positive bacteria than against Gram-negative bacteria, like most temporin AMPs [13]. Based on the online tools for peptide analysis, in comparison with most temporin peptides charged with lysine, GHa is positively charged by histidine. In order to investigate how the different positively-charged amino acid residues affect the antimicrobial activity of temporin peptides, lysine was used to replace histidine at both ends of GHa to design the single-point or multi-point mutation peptides. Three derived peptides were obtained—GHaK, GHa4K, and GHa11K. The antibacterial activities of the derived peptides against Gram-negative and Gram-positive bacteria were enhanced. We also investigated the bactericidal mechanism and the effect of those peptides on *S. aureus* biofilm. In addition, the hemolytic activity of these four peptides in the presence or absence of *S. aureus* was determined.

## 2. Results

### 2.1. Statistical Analysis of Bioinformatics

After decades of research, more and more natural and designed AMPs have been reported, and various antimicrobial peptide databases have emerged in the era of big data, providing researchers with very convenient and powerful tools to learn more about the AMPs. The DBAASP database was used to calculate the length, net charge, isoelectric point and hydrophobic moment tendency of AMPs [14]. It is found that most of the AMPs are 6–20 amino acids in length (Figure S1A), with the net charge in the range of +2–+7 (Figure S1B). The isoelectric point is in the range of 7.6–11.6 (Figure S1C), and the hydrophobic moment is about 1.2–1.9 (Figure S1D). GHa and its derived peptides are composed of 13 amino acid residues with a net charge of 1–2. Physicochemical properties of those four peptides are consistent with the common properties of AMPs (Table 1), indirectly indicating that they may have antimicrobial activity.

In order to design a derived peptide with enhanced antibacterial and antibiofilm activity, we performed the bioinformatics statistics on different groups of AMPs by using APD3 [15]. The results show that the high frequency amino acids in the AMPs are the hydrophilic amino acid G, the hydrophobic amino acid L and the positively-charged amino acid K and R, whereas the positively-charged amino acid H has a very low frequency (Figure S2A). This is particularly pronounced in the temporin family (Figure S2B) and antibiofilm peptides (Figure S2C). In temporins, the dominant hydrophobic amino acids are leucine (29.86%), phenylalanine (11.72%), and isoleucine (10.64%). Hydrophilic amino acids include glycine (10.33%) and serine (6.95%). For basic amino acids, temporins typically use lysine (6.97%) instead of arginine (1.14%) and histidine (0.9%). There are no cysteines in temporins, and the acidic amino acids glutamate and aspartate are rare. We also analyzed the amino acid frequencies of the 122 temporin family antimicrobial peptides by using WebLogo (Figure S2D) [16]. WebLogo is a web-based application designed to make the sequence logos as easy and painless as possible. Sequence logos are a graphical representation of an amino acid or nucleic acid multiple sequence alignment. Each logo consists of stacks of symbols, one stack for each position in the sequence. The overall height of the stack indicates the sequence conservation at that position, while the height of symbols within the stack indicates the relative frequency of each amino at that position. The results showed that the amino acids at the 1st, 2nd, 3rd, 9th, 12th, and 13th positions were more conservative occurring with F, L, P, L, L, and L. The positively-charged amino acid K has the highest frequency at positions 7 and 11, and the C-terminus is more likely to be positively charged than the N-terminus [17]. These statistical results have greatly inspired our design ideas for new AMPs.

### 2.2. Peptide Design and Physicochemical Properties

As the number of temporins identified in nature increases, it is useful to conduct bioinformatics analysis to gain insight into the general design principles of natural temporins. Interestingly, GHa contains H at the 4th and 11th positions, which is a low frequency positively charged amino acid in the most of temporins. The difference stimulated our curiosity about how the positively-charged amino acid affects the antimicrobial activity in temporins. Therefore, lysine was used to replace histidine at both ends of GHa to design the single-point or multi-point mutation peptides, and obtained the following three derived peptides (Table 1). The physicochemical properties of the peptides were analyzed, which might also provide us with a clue to their bioactivity. Compared to the GHa, the hydrophobic moment (μH) of the derived peptides was increased and the amphiphilicity enhanced, which was caused by the substitution H (the hydrophilic value of −3.2) with K (the hydrophilic value of −3.9), and the smaller hydrophilic value, the better hydrophilicity. As a general rule, the α-helical AMPs with stronger activity are associated with higher amphiphilic and higher hydrophobic moment and they have a positive grand average hydropathy (GRAVY) and negative or close to the 0 Boman index (BI) [18]. The GRAVY value of the derived peptides was decreased, and the charge, Boman index, hydrophobic moment, and amphipathicity were increased. Changes in these properties may enhance the activity of the designed peptide.

### 2.3. The Activity and Structure Prediction

The prediction tools of AMPs bioactivity allow to conduct task-oriented design of the new AMPs and consequently diminish the costs of aimless production. In order to test the rationality and feasibility of the design ideas, we used the antimicrobial peptide prediction tool to predict the activity of GHa and its derived peptides. The results of the four prediction models of CAMP_R3_ were positive, and the probability scores predicted by the three models of SVM, RF, and DA indicated that the activity of the analogs was stronger than the GHa, especially GHaK and GHa11K (Table S1) [19]. As shown in Table S2, all the derived peptides were active against Gram-positive bacteria *S. aureus* and *B. subtilis*. Furthermore, the antimicrobial spectrum of GHaK and GHA11K was extended to Gram-negative bacteria *E. coli* and *P. aeruginosa*. However, the GHa only showed the antibacterial activity against *S. aureus*. In addition, the derived peptides were predicted to have stronger antibiofilm activity than GHa.

**Figure 1:**
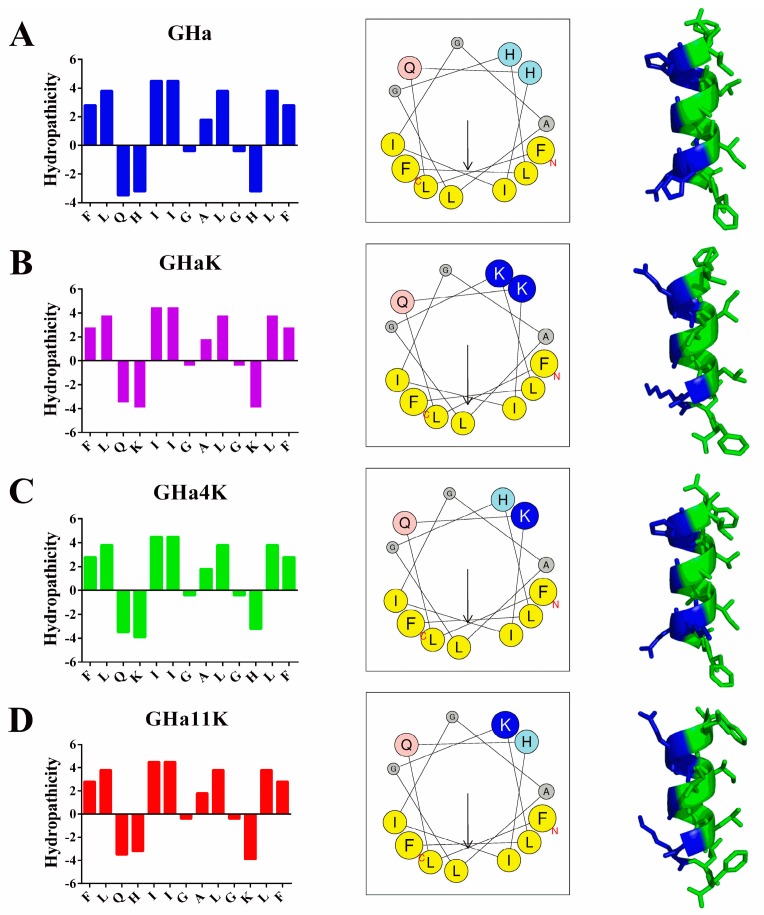
The distribution of hydrophilic and hydrophobic amino acid residues, helical wheel projection and the predicted 3D structures. The four rows of (**A**), (**B**), (**C**), and (**D**) are GHa, GHaK, GHa4K, and GHa11K. The left column showed the amphipathic profile calculated by the ExPASy. The middle column showed the helical wheel projection of the peptides generated by using the Heliquest (http://heliquest.ipmc.cnrs.fr/). The arrows indicated the direction of the hydrophobic moment. The right column demonstrates the 3D structure, analyzed by using PEP-FOLD (http://bioserv.rpbs.univ-paris-diderot.fr/services/PEP-FOLD/), with a hydrophilic surface in blue and a hydrophobic surface in green.

The helical wheel projection by using Heliquest showed that the hydrophilic amino acid residues of the peptides were on one side and the hydrophobic ones on the other side of the helix, contributing to the large hydrophobic face for the peptides [20]. Consistent with the prediction results, the predicted 3D model of the peptides by using PEP-FOLD also shows an α-helix structure with hydrophilic and hydrophobic amino acids on each side, presenting a perfect amphiphilic structure (Figure 1) [21]. The increased hydrophobic moment also indicated that the derived peptides possessed a better balance between the hydrophobic phase and the hydrophilic phase than the GHa [22].

### 2.4. Derived Peptides Showed Higher and Broader Spectrum Antibacterial Activity Than the Parent Peptide

The antimicrobial activity of GHa and its derived peptides against tested strains was summarized in Table 2. The derived peptides had a more efficient and broader spectrum of antibacterial activity than the GHa which only showed less activity against *Staphylococcus aureus*, *Streptococcus mutans* and *Escherichia coli*, and no antifungal activity. The MICs of the derived peptides were reduced by 4–8 times. GHaK displayed potent antimicrobial activity with the MICs of 1.6–25 μM against both Gram-positive and Gram-negative bacteria. The derived peptides also obtained the antibacterial activity against the drug-resistance stain, methicillin-resistant *Staphylococcus aureus* (MRSA) with the MICs in the range of 6.2–12.5 μM. The double site mutant GHaK showed similar high antimicrobial activity to that of the C-terminal single site mutant GHa11K, while the N-terminal single mutant GHa4K had a relatively weak activity. The MBCs of the derived peptides were close to the MICs value, and reduced by 4–32 times, indicating that the derived peptides exerted a stronger bactericidal action than the GHa. In contrast, the bactericidal activities of the derived peptides were more powerful than that of kanamycin. In addition, the antifungal activity of GHaK was stronger than that of GHa, and GHa11K was similar to GHa. Surprisingly, GHa4K lost antifungal activity. The results of the antimicrobial activity assay were consistent with the predictions of CAMP_R3_ (Table S1), while only GHaK was consistent with the predictions of DBAASP (Table S2). Overall, these predictions are still of great value. Considering that the derived peptides showed the highest antibacterial activity against *S. aureus*, it was utilized for the following experiments.

### 2.5. Growth Inhibition Kinetics

**Figure 2:**
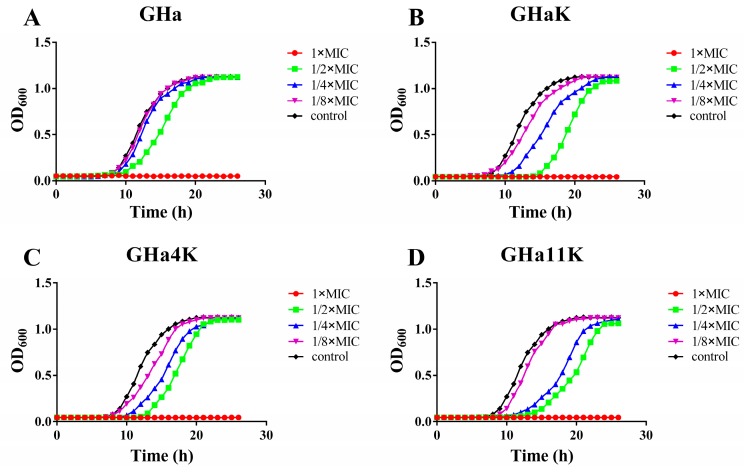
Growth inhibition kinetics of (**A**) GHa, (**B**) GHaK, (**C**) GHa4K, and (**D**) GHa11K. The growth of *S. aureus* (OD600) was grown in TSB broth for 26 h in the presence of 1 × MIC, 1/2 × MIC, 1/4 × MIC and 1/8 × MIC peptides, with PBS as the control.

We evaluated the effect of sub-MIC levels of peptides on the basic viability of *S. aureus* by growth curve assay. As shown in Figure 2, the growth curve of *S. aureus* includes three phases: a lag phase, an exponential phase, and a stationary phase. We analyzed the time to reach the stationary phase and lag phase of the growth curves (Table S3). When treated by GHa and its analogs at 1 × MIC, the growth of *S. aureus* was completely inhibited. When treated with 1/4 × MIC and 1/8 × MIC GHa, the growth of *S. aureus* was almost unaffected, and the growth kinetics of the treatment group were the same as the control; they started the logarithmic phase after 10 h, plateaued around 20 h, and stepped into the stationary phase. The lag phase was delayed for approximately 2 h by GHa at 1/2 × MIC. In contrast, after treated by the derived peptides treatment at a low concentration of 1/8 MIC, *S. aureus* exhibited an extended (1 h) lag phase and lower growth during the logarithmic phase. However, the time period to reach a stationary phase was still 20 h. At concentrations of 1/2 × MIC and 1/4 × MIC, the inhibitory effect of the derived peptides on the bacteria was more significant, which the lag phase was delayed by 2–7 h and the time to reach the stationary phase was also extended by 3–4 h. However, in the stationary phase, the average absorbance of all peptide treatments was not significantly different from that of the control group. The results showed that the growth inhibitory effect of the derived peptides on *S. aureus* was much higher than that of the GHa.

### 2.6. Killing Kinetics of GHa and Its Derived Peptides

**Figure 3:**
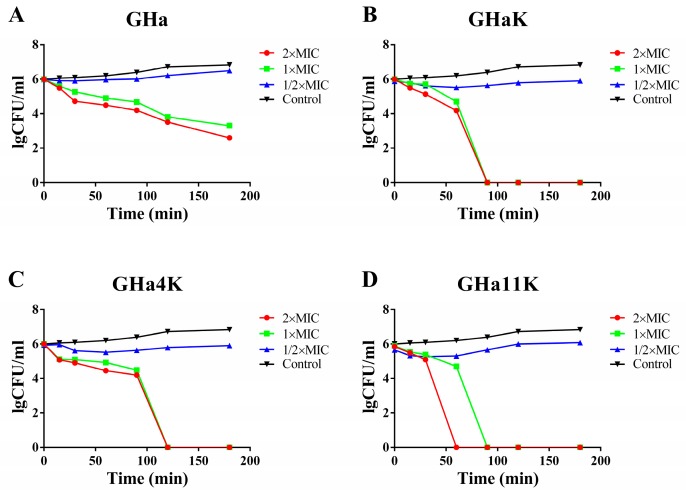
Killing kinetics of *S. aureus* after treatment with (**A**) GHa, (**B**) GHaK, (**C**) GHa4K, and (**D**) GHa11K at 1/2 ×MIC, 1 × MIC and 2 × MIC.

The time killing curves of GHa and its derived peptides against *S. aureus* were plotted in Figure 3. All peptides showed the concentration-dependent and time-dependent bactericidal activity against *S. aureus*. The bacteria were all killed within 90 min for GHaK at the concentrations of 2 × MIC and 1 × MIC (Figure 3B), and 120 min for GH4K (Figure 3C). GH11K completely killed all bacteria in 90 min at a concentration of 1 × MIC, and 60 min at a concentration of 2 × MIC (Figure 3D). In contrast, GHa did not completely eradicate the bacteria even after 180 min with the bacterial number decreased by approximately three orders of the magnitude (Figure 3A). The results of killing kinetics was consistent with the MBC values and the derived peptides showed a faster bactericidal efficiency than the GHa.

### 2.7. Effect of GHa and Its Derived Peptides on the Bacterial Membrane Permeability

**Figure 4:**
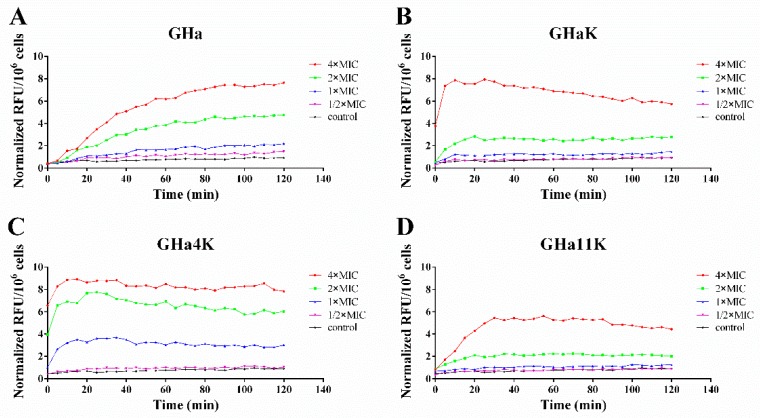
The membrane permeability assay on *S. aureus* by fluorescence spectroscopy. Bacteria were treated by (**A**) GHa, (**B**) GHaK, (**C**) GHa4K, and (**D**) GHa11K at various concentrations. Normalized RFU: normalized relative fluorescence units.

We compared the membrane permeability of GHa and its analogs by fluorescence spectroscopy in the presence of a probe molecule. We conducted the fluorescence experiment simultaneously with the bacterial growth inhibition experiment. The results showed that *S. aureus* did not proliferate within two hours, and the total number of cells was 10^8^ CFU/mL in each treatment (Figure S3). When the bacterial membranes are damaged, the membrane-impermeable propidium iodide (PI) will enter the bacteria and bind to its DNA, leading to a rapid increase in fluorescence. In order to make the different data comparable to each other, the relative fluorescence units (RFU) was normalized by the total number of cells. As shown in Figure 4, the PI fluorescence in both GHa and its derived peptides treatments increased in a concentration-dependent and time-dependent manner. After the bacteria were treated with the peptides at 4 × MIC, the maximum fluorescence was increased by about 15–20 times compared with the negative control. In comparison with GHa reaching the maximum normalized RFU at 90 min, the derived peptide reached to the maximum normalized RFU faster. Both GHaK and GHa4K reached to the maximum at 10 min, and 30 min for GHa11K. These results indicated that all peptides interacted with bacterial membranes to exert their antibacterial activity and the membrane permeability of the derived peptide were stronger.

### 2.8. Effects of GHa and Its Analogues on Bacterial Initial Attachment, Biofilm Formation, and Preformed Biofilms

**Figure 5:**
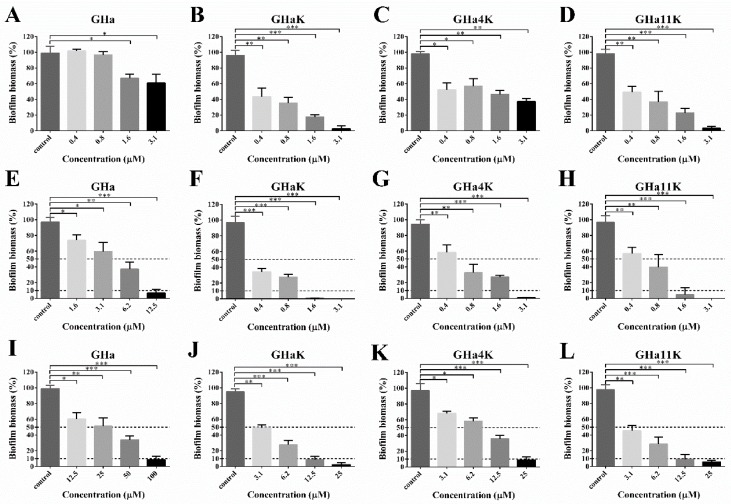
The biofilm attachment inhibitory of the (**A**) GHa, (**B**) GHaK, (**C**) GHa4K, and (**D**) GHa11K, biofilm formation inhibitory of the (**E**) GHa, (**F**) GHaK, (**G**) GHa4K, and (**H**) GHa11K and biofilm eradication of the (**I**) GHa, (**J**) GHaK, (**K**) GHa4K, and (**L**) GHa11K. Dashed lines represent the 10% and 50% of the biofilm biomass (* *p* < 0.05; ** *p* < 0.01; *** *p* < 0.001).

Surface attachment is considered to be the first step in biofilm formation. The inhibition of GHa and its derived peptides on initial bacterial adhesion of *S. aureus* was tested (Figure 5A–D). The derived peptides GHaK and GHa11K showed a dose-dependent inhibitory activity with the inhibition rate of 51–82.5% at the concentration of 0.4–1.6 μM, and completely inhibited bacterial adhesion at a concentration of 3.1 μM. GHa4K inhibited 62.6% initial attachment at 3.1 μM. In contrast, GHa had only an inhibitory effect of 39.2% at the highest concentrations of 3.1 μM, and no obvious effect at low concentrations (0.4–0.8 μM). Overall, the derived peptides were more efficient to inhibit the initial adhesion of the bacteria than GHa.

The ability of peptides to prevent biofilm formation presented by the peptide’s minimum biofilm inhibitory concentration (MBIC) was investigated [23]. The MBIC is defined as the lowest concentration of an antimicrobial agent required to inhibit the formation of biofilm. As shown in Figure 5E–H), GHa and its analogs were found to be potent inhibitors of biofilm formation of *S. aureus* in a dose-dependent inhibitory manner. MBIC required to inhibit the formation of 50% of biofilm (MBIC_50_) or 90% of biofilm (MBIC_90_) were shown in Table 3. The MBIC_50_ and MBIC_90_ of GHa are 6.2 μM and 12.5 μM, respectively. The MBIC_50_ value of the derived peptides was reduced by 8–16 times, and the MBIC_90_ value was reduced by 4–8 times. The ability of the derived peptides to inhibit the formation of *S. aureus* biofilm was significantly improved.

The activity of GHa and derived peptides to eradicate the preformed biofilms was determined and presented by the peptide’s minimum biofilm eradication concentration (MBEC) [23]. The MBEC is defined as the lowest concentration of an antimicrobial agent required to eradicate the formed biofilm. As shown in Figure 5I–L, all peptides exerted a dose-dependent eradication ability to the biofilm. GHaK and GHa11K showed an efficient ability to eliminate mature biofilms with an eradication rate of more than 50% at a concentration of 3.1 μM. When the concentration of these two peptides was up to 25 μM, almost all the biofilms were disrupted. MBEC required to eradicate 50% of the formed biofilm (MBEC_50_) or 90% of the formed biofilm (MBEC_90_) were shown in Table 3. The MBEC_50_ and MBEC_90_ of GHaK and GHa11K were both 3.1 μM and 12.5 μM, and 12.5 μM and 25 μM for GHa4K. However, GHa’s MBIC_50_ and MBEC_90_ were as high as 50 μM and 100 μM. Therefore, these results showed that the derived peptides exhibited the enhanced ability to inhibit bacterial adhesion, biofilm formation, and to eradicate the mature biofilms of *S. aureus* compared to the GHa.

### 2.9. Hemolytic Activity of Peptides

**Figure 6:**
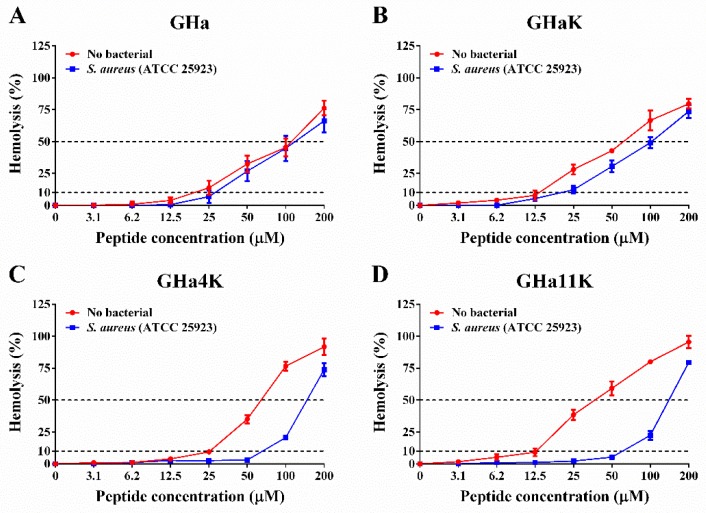
Hemolytic activity of (**A**) GHa, (**B**) GHaK, (**C**) GHa4K, and (**D**) GHa11K in the absence and presence of *S. aureus*. Dashed lines represent 10% and 50% hemolysis. Hemolysis values ≤10% are considered to be non-hemolytic. Data are reported as mean ± standard error of three independent experiments. The red line represents bacteria-free, and the blue line represents the hemolytic activity in the presence of *S. aureus*.

The therapeutic index (TI) is the ratio of the MHC to the geometric mean of the MICs (GM) and cell selectivity index (CSI) is the ratio of HL50 to the MIC of the peptide against *S. aureus* (ATCC 25923). Larger values indicate greater cell selectivity. The TI and CSI were used to evaluate the selectivity of peptides against the bacterial membrane versus the zwitterionic mammalian membranes [24,25]. Based on the results of the hemolytic activity in Figure 6, we calculated the minimum hemolytic concentration that caused 10% hemolysis (MHC), the concentration at which 50% of human red blood cells (hRBCs) were lysed (HL_50_), as well as CSI and TI (Table 4). The TI of GHa was 0.5, while the GHaK, GHa4K, and GHa11K were 2.8, 3.7, and 2.3 respectively, which increased by 4–8 times. The CSI of the derived peptides was also increased by 2–4 times compared to the GHa, and the CSI of GHaK was as high as 42.3. In the presence of *S. aureus*, the HL_50_ of the derived peptides was significantly increased, while the hemolytic activity of GHa was unchanged, which also indicated that the selectivity of the derived peptides to bacteria was greatly enhanced. It was very interesting that the derived peptides with a better selectivity index were more effective than the parent peptide in killing *S. aureus*. These results indicated that the derived peptides preferred to bind with bacterial cells rather than to human red blood cells (hRBCs), which implied a broader therapeutic window of the derived peptides.

## 3. Discussion

Due to the global prevalence of antibiotic-resistant infections and the rapid decline in the discovery of new antibiotics, exploring the new antibacterial agents have become an urgent problem to be solved [26]. In the past two decades, AMPs have been considered as the promising antibacterial drug candidates which may change the treatment of infectious diseases [27], because of their broad-spectrum antibacterial activity, rapid mode of action, and the very low potential to induce resistance. However, we still face the tremendous challenges of their clinical application [25]. Compared with the conventional medical antibiotics, AMPs exhibit relatively low antimicrobial activity, as well as the poor oral availability, the degradation by proteases, the high hemolysis, lack of specific target et al. Molecular design of AMPs is one of the possible strategy to improve the antimicrobial activity, stability, and reduce the hemolysis for the development of new AMPs. Naturally-occurring AMPs from a variety of natural sources, such as plants, animals and humans, provide rich compositional and structural information to design the novel derived AMPs with enhanced antimicrobial activity and cell selectivity [28]. Meanwhile, the production costs are reduced due to the shorter sequences and the improved stability. Nowadays, template-based design, de novo minimalist design, and the use of bioinformatics and combinatorial libraries are generally used in the design of novel AMPs [28]. Most AMPs used for in vitro studies are chemically synthesized, which contain peptide, counter-ion, and water. The peptide secondary structure and antimicrobial activity might be influenced by the different counter-ions [29]. The peptide salt of trifuoroacetate (TFA) showed slightly less antimicrobial activity than acetate or chloride salts. However, the high CSI (1.36) was obtained in temporin A TFA salts, and no selectivity observed in temporin A acetate and chloride salts [29]. Thus, in this study, we synthesized temporin TFA salts.

The template-based design preserves the evolutionary conservation of the peptide sequences and reduces the number of candidate peptides synthesized to obtain an effective AMP. In this study, we combined the template-based design with the database-assisted design to obtain the derived AMPs with highly effective antibacterial and antibiofilm activity. The GHa cloned by our laboratory was used as a template for designing a series of analogs. GHa is similar to most temporin peptides such as temporin 1Tb, temporin-PE [30,31], and showing potent activity against Gram-positive bacteria instead of Gram-negative bacteria. In order to improve the therapeutic potential of GHa, we designed three derived peptides GHaK, GHa4K, and GHa11K. The derived peptides showed more efficient antimicrobial activity than GHa, and obtained the broader spectrum against both Gram-positive and Gram-negative bacteria. In addition, GHaK and GHa11K also had the antifungal activities. The antibacterial activity of the derived peptides was 4–8 times higher than the parent peptide, 2–4 times stronger than temporin-SHd [32]. Interestingly, the amino acid substitution K for H made the derived peptides obtain the antibacterial activity against the drug-resistance strain, MRSA. The antibacterial activity of the two-site mutant GHaK was similar to that of the C-terminal single point mutant GHa11K, while that of the *N*-terminal single mutant GHa4K was relatively weak. We deduced that the C-terminus of temporin peptides plays an important role in the peptides displaying the antimicrobial activity, in which K at 11th position is the most crucial amino acid. Lysine residues in the peptides contribute mostly to improve the antimicrobial activity of AMPs rather than histidine, which might be due to the fact that the electrostatic driving force between lysine and the negatively-charged phospholipid of the bacterial membrane is stronger than histidine. Therefore, in the course of centuries of species evolution, high-frequency amino acids existing in AMPs were supposed to provide a vital role for the survival of living organisms.

In addition, a large amount of direct and indirect evidence indicates that the cation-π interaction is important in various proteins that bind to cationic ligands or substrates [33,34]. The cation-π interaction may occur between lysine and phenylalanine in the derived peptide (F at the 1st position and K at the 4th position or F at the 13th position and K at the 11th position). This promotes deeper membrane insertion of the peptides, thereby increasing the antibacterial activity [33]. Interestingly, the derived peptides also enhanced the activity against Gram-negative bacteria. AMPs with antibacterial activity against Gram-negative bacteria tend to have more net charges than those against Gram-positive bacteria [35]. We analyzed the peptides in APD3 [15], and found that the average net charge of AMPs with the antibacterial activity against Gram-negative bacteria was 3.49, while those against Gram-positive bacteria was 2.35. Furthermore, the cell wall of the Gram-negative bacterial consists of a peptidoglycan layer and an outer membrane layer which is mainly composed of anionic lipopolysaccharide (LPS) [36]. Therefore, electrostatic interactions play the crucial role in the peptide-bacteria interactions. According to Rosenfeld et al., in addition to the low positive charge, the inactivity of temporins A and B against Gram-negative bacteria was that the peptides oligomerized when they were in contact with the outer membrane LPS [36,37]. Such aggregation was unable to efficiently diffuse through the bacterial cell wall to reach and penetrate the plasma membrane due to large volume. We deduced that the enhanced activity of GHa derived peptides against Gram-negative bacteria caused by the replacement of histidine by lysine, which increases the net positive charge of the peptides. The peptides showed the stronger repulsive force with each other and are not easily oligomerized while contacting with LPS, thereby diffusing through the bacterial cell wall to reach and penetrate the plasma membrane. The hydrophobic moment is positively correlated with the efficient membrane-perturbing action of AMPs [38]. The substitutions of basic amino acid in GHa derived peptides contribute them to high hydrophobic moment compared with GHa, which also enhances their antibacterial activity. However, the derived peptide’s activity against Gram-negative strains is lower than Gram-positive strains, which is still needed to be study further. In comparison with other AMPs, temporins are amongst the shortest naturally occurring AMPs, containing only a few basic amino acid residues (the net positive charge ranging from 0 to +3), which might be one of the reasons they possess low activity against Gram-negative bacteria [38].

Previous studies have shown that only membrane-active AMPs such as daptomycin and human cathelicidin-derived 17BIPHE2 can target bacterial membranes rather than non-membrane-targeted AMPs [39]. The results of the membrane permeability of GHa and its mutants indicated that they targeted the bacterial membranes to exert their antibacterial activity. The rate of damage on the bacterial membrane caused by the derived peptides was faster, which explained why they were more active than the parent peptide. The results of Falanga et al. was consistent with ours, and the modification of the peptide GH625 with a series of lysine residues improved microbial membrane permeation ability [40].

Biofilm formation usually involves the initial attachment of bacteria to the surface, growth and maturation stages [41]. Extracellular polymeric substance (EPS) produced by bacteria is the main extracellular matrix in biofilms, which has been reckoned as a diffusion barrier to antimicrobial agents [42]. Preventing antibiotic from penetrating into the biofilm may play an important role in the drug resistance. Vancomycin can diffuse through the biofilm extracellular matrix of *S. aureus*, causing the bacteria in deep biofilm death [43]. PTP-7 (FLGALFKALSKLL) derived from Gaegurin 5, is a positively-charged lytic peptide, which penetrated *S. aureus* biofilms to approach the deep layer, and killed bacteria inside biofilms efficiently [44]. GHa derived peptides showed a significantly enhanced ability to inhibit the adhesion and formation of *S. aureus* biofilm, as well as to disrupt the mature biofilm by 4–8 times compared to the parent peptide. The molecular weight of GHa derived peptides is roughly equivalent with vancomycin, and the peptide sequences of GHa derived peptides are homologous with PTP-7, as well as hydrophobicity. So, we deduced that the derived peptides effectively penetrated the biofilm and killed the bacteria. Considering that the currently used antibiotics such as tetracycline, tobramycin, and norfloxacin can induce biofilm formation at a level of sub-MIC leading to exacerbating the severity of infections [45], these derived peptides will be the promising antibiofilm agents.

Evaluation of cytotoxicity is a necessary step in the development of peptide-based antimicrobial agents. The delicate balance between the cations, amphiphilicity, hydrophobicity, and helicity of AMPs determines the antimicrobial potency and cell selectivity [46]. We tested the hemolytic activity of the peptides in the presence and absence of *S. aureus*. GHa and derived peptides showed lower hemolytic activity than temporin 1Ga (HL_50_ = 12.5 μM), temporin-PE (HL_50_ = 39.64 μM) and melittin (HL_50_ = 2.5 μM) [24,31,47]. Moreover, the TI of the derived peptides increased by 4–8 times and the CSI increased by 2–4 times. In the presence of *S. aureus*, the HL_50_ of the derived peptides increased significantly, while the hemolytic activity of GHa did not change. These results indicated that the selectivity of the derived peptides to bacteria was greatly enhanced, and they preferred to bind to bacterial cells rather than to hRBCs, which means a broader therapeutic window of the derived peptides. The superior cell selectivity makes the analogs the promising new antimicrobial agents for the treatment of *S. aureus* infections.

In summary, the derived peptides showed stronger antimicrobial and antibiofilm activity than the GHa. The substitution of K for H increased the positive charges of the derived peptide, leading to a stronger and faster bactericidal activity and a broader-spectrum of action, as well as the enhanced bacterial membrane permeation. In addition, the GHa analogs showed stronger potent inhibition on the biofilm adhesion and formation, and also can eradicate the mature biofilms of *S. aureus*. The superior cell selectivity makes the analogs the promising new antimicrobial agents for the treatment of *S. aureus* infection.

## 4. Materials and Methods

### 4.1. Strains and Growth Conditions

Bacterial strains were used to determine the antimicrobial activity of temporin peptides, including the Gram-positive bacteria *Staphylococcus aureus* (ATCC 25923), *Streptococcus mutans* (ATCC 25175), *Bacillus subtilis* (ATCC 6633), methicillin-resistant *S. aureus* (ATCC 43300), methicillin-resistant *S. aureus*-2 (isolated), and the Gram-negative bacteria *Escherichia coli* (ATCC 25922), *E. coli* (D31), *Pseudomonas aeruginosa* (ATCC 15442), *P. aeruginosa* PAO1 (wild type), as well as fungi *Candida albicans* (ATCC 10231). Bacterial strains were grown in Tryptic Soy Broth (TSB) (HuanKai Microbial, China). For the colony-forming units (CFU) count, serially diluted bacterial suspensions were plated on Tryptic Soy Agar (TSA) (HuanKai Microbial, Guangzhou, Guangdong, China) and incubated for 24 h at 37 °C. The fungi were grown in Sabouraud Dextrose Broth (SDB) (HuanKai Microbial, China). All experiments were conducted using bacterial cells and *C. albicans* in the logarithmic-phase of growth (OD_600_ = 0.4–0.6).

### 4.2. Bioinformatics Statistical Analysis, Peptide Design and Physicochemical Properties

A combination of template-based design and database-aided design method was used to design novel antimicrobial peptides. Briefly, the bioinformatics methods were used to collect and combine statistical data from different antimicrobial peptide databases, and the APD3 was used to analyze the amino acid profile of the antimicrobial peptide, the temporin family, and the antibiofilm peptides [15]. The length, net charge, isoelectric point, and hydrophobic moment tendency of antimicrobial peptides were counted using the DBAASP [14]. In addition, the amino acid frequencies of each site of the 122 temporin family antimicrobial peptides were analyzed using WebLogo [16]. Combined with the above statistical results, a series of peptide analogues were designed using the antibacterial peptide GHa of *H. guentheri* as template. Then, the physicochemical properties of peptides were analyzed using the DBAASP and the APD3.

### 4.3. Activity and Structure Prediction

Three different antimicrobial peptide prediction tools were used; the AMP prediction tool of the CAMP_R3_, the DBAASP and the dPABBs [48]. CAMP_R3_ combines amino acid sequence composition, physicochemical properties, and structural features using four different predictive models, support vector machine (SVM), random forest (RF), artificial neural network (ANN) and discriminant analysis (DA). Therefore, the SVM, RF, and DA models give probability scores (between 0 and 1). Higher scores mean that the peptide is more likely to exert antimicrobial activity. The results predicted by the ANN model are as follows. AMP: predicted to be an antibacterial sequence. NAMP: a sequence that is not expected to have an antibacterial effect. The DBAASP is particularly useful in predicting the activity of peptides against *S. aureus*, *E. coli*, *P. aeruginosa*, and *B. subtilis*. The dPABBs was used to predict the activity of antibiofilm by SVM and WEKA-based models. The PEP-FOLD server was used to predict the 3D model of the peptides. A 3D model of the peptide was presented using the visualization software PyMol. The hydropathicity profile was calculated with the amino acid hydropathicity scale through the ExPASy web server [49]. Heliquest was used to predict the helix diagram of the peptides.

### 4.4. Synthesis of Peptides

GHa and analogs (Table 1) were synthesized by GL Biochem (Shanghai, China) using the solid-phase with the N-9-fluorenylmethyloxycarbonyl (Fmoc) strategy and purified by high-performance liquid chromatography (HPLC). A preparative liquid chromatograph (LC6000) was used for preparation and a high-performance liquid chromatograph (LC3000) for analysis. Preparation conditions: the detection wavelength is 220 nm, the flow rate is 180 mL/min and preparation column is C18 column. The mobile phase included 0.1% TFA in 100% acetonitrile and 0.1% TFA in 100% water. C-terminals of the synthetic peptides were modified by amidated. The identity of each peptide was confirmed by mass spectrograph. Peptide purity used in biologic assays was higher than 95%. The peptides were dissolved in water for a 2 mM stock solution for further use.

### 4.5. Minimum Inhibitory Concentration (MIC) and Minimum Bactericidal Concentration (MBC) Assays

In brief, different concentrations of the peptides and antibiotic were incubated with microorganisms under defined conditions. The microbial suspension was diluted with fresh medium to a concentration of 2 × 10^6^ CFU/mL. Peptides and antibiotic were dissolved in the culture medium and then 50 μL of each peptide and antibiotic solution was mixed with 50 μL of diluted bacterial suspensions to get a final concentration range from 0.8–100 μM in two-fold dilution. The wells not treated with antimicrobial agents served as the positive control and the medium without bacteria inoculation as the negative control. After incubation at 37 °C for 24 h, the absorbance of each well was recorded using a multi-well microplate reader (Multiskan Spectrum, Bio Tek, Winuski, VT, USA) at 600 nm. The lowest concentration at which the peptide inhibited the growth of the bacteria completely was taken as the MIC. MBC were determined by plating 50 μL of bacterial suspensions from the MIC assay on and above the MIC values on TSA. Plates where no bacterial growth was visible after incubation at 37 °C for 24 h were considered to be the MBC.

### 4.6. Growth Curve Assay

Growth inhibition kinetics of peptides on *S. aureus* was determined as described previously [50]. Aliquots of overnight culture of *S. aureus* were diluted in TSB broth to obtain the final concentration of 1 × 10^6^ CFU/mL. Peptides was then added into *S. aureus* culture to the final concentrations of 1/8 × MIC, 1/4 × MIC 1/2 × MIC and 1 × MIC. Sterile TSB acted as a blank control. These plates were incubated at 37 °C. The absorbance at 600 nm (OD_600_) was determined using a microplate spectrophotometer (Multiskan Spectrum, Bio Tek, Winuski, VT, USA) every hour throughout 26 h incubation.

### 4.7. Time-Killing Curves

Kinetics of the bacterial killing was measured following the previously described method [30]. The peptides were prepared in two-fold serial dilution with TSB in Eppendorf tubes. Then, 300 μL of diluted bacterial suspension (final concentration of 1 × 10^6^ CFU/mL) were added to each Eppendorf tube to make the peptides at the final concentrations of 1/2 × MIC, 1 × MIC and 2 × MIC. Tubes were incubated at 37 °C, and an aliquot was collected at 0, 15, 30, 60, 90, 120, and 180 min for plating on the TSA to determine the viable counts. Serial dilutions (10^−1^–10^−4^) of the aliquots were prepared in TSB. The diluted samples (100 μL) were plated onto TSA and incubated at 37 °C for 24 h. The number of colonies was determined. Killing curves were constructed by plotting the log10 CFU per milliliter versus time.

### 4.8. Membrane Permeation by Fluorescence Spectroscopy

The experiment was performed as described [18]. Briefly, 100 μL of serially diluted 2 × peptide was prepared in 96-well corning COSTAR microtiter plates. Then, 8 μL of propidium iodide (the final concentration of 20 μM) and 92 μL of the *S. aureus* bacterial culture (the final density of 1 × 10^8^ CFU/mL) were added in each well. The plates, with continuous shaking at 100 rpm at 37 °C in a Hybrid multi-mode microplate reader (Synergy H1, Bio Tek, Winuski, VT, USA), were read every 5 min during 2 h. The excitation and emission wavelengths for fluorescence measurement were set at 584 nm and 620 nm, respectively.

### 4.9. Antibiofilm Assay

#### 4.9.1. Inhibition of bacterial initial attachment

The inhibition of the peptides on biofilm attachment of *S. aureus* was detected. Overnight cultures of *S. aureus* were resuspended in TSB (containing 1% glucose) to a concentration of 2 × 10^8^ CFU/mL. Aliquots of 100 μL suspended bacteria were added to a 96-well plate containing 100 μL peptides in TSB by two-fold dilution, and the peptide-free were served as a negative control. The plate was incubated at 37 °C for 4 h without agitation to allow bacterial attachment and the biomass of *S. aureus* biofilm was examined with 3-[4,5-dimethylthiazol-2-yl]-2,5-diphenyltetrazolium bromide (MTT) assay as below.

#### 4.9.2. Inhibition of biofilm growth

Briefly, *S. aureus* were grown to the logarithmic phase in TSB at 37 °C and diluted in TSB (with 1% glucose) to obtain a concentration of 2 × 10^6^ CFU/mL. *S. aureus* suspensions were inoculated into 96-well microplates in the absence (negative control) or in the presence of different concentrations of each peptide. Then the plates were incubated at 37 °C for 24 h and the biomass of *S. aureus* biofilm were examined with MTT assay as below.

#### 4.9.3. Preformed biofilm disruption

To evaluate the biofilm-disrupting property, *S. aureus* (10^6^ CFU/mL) was made from exponentially growing bacteria in fresh TSB media. Two hundred microliters inoculum culture were seeded to a 96-well-flat-bottom plate and incubated at 37 °C for 24 h to obtain the mature biofilm. Afterwards, the plate was washed by sterile phosphate buffered saline (PBS) twice and treated with a series 200 μL peptide solution at 37 °C for 24 h. The ability of peptide disruption of biofilms was quantified with MTT assay as below.

#### 4.9.4. Biofilm metabolic activity assay (MTT assay)

The metabolic activity of the biofilms formed by the bacteria was assessed using a modified MTT assay [51]. Briefly, MTT was dissolved in PBS to 5 mg/mL. The culture medium in the plates treated as above was removed gently and the plates were air-dried. Five microliter of 5 mg/mL MTT solution and 95 µL of PBS (pH 7.2) was added to each well and incubated for 3 h at 37 °C. The insoluble purple formazan was further dissolved in 150 μL dimethyl sulfoxide (DMSO). The absorbance was measured at 570 nm using the microplate reader. The percentage inhibition of metabolic activity was calculated as: [(OD_570_ control without peptide − OD_570_ peptide)/OD_570_ control without peptide] × 100%.

### 4.10. Hemolytic Assays

The hemolytic activity of the peptides was determined as described [24]. Human red blood cells were washed at 1000 g for 10 min at 4 °C with PBS (pH 7.2) 3–5 times, until the supernatant was clear. A final of 2 × 10^8^ cells/mL (4%) hRBC was resuspended in PBS (pH 7.2). The aliquot 300 μL of hRBC resuspension and 300 μL of serially diluted peptide were mixed in 1.5 mL tube and incubated at 37 °C for 60 min. After incubation, the mixture was centrifuged at 1000× *g* for 10 min to collect the supernatant. A total of 150 μL of the supernatant were removed to a new 96-well plate and the absorbance was detected at 450 nm. The absorbance of PBS treated-cell represented zero hemolysis, and the 0.1% Triton X-100 treated cell represented 100% hemolysis. In addition, peptide hemolysis was evaluated by incubating hRBCs solutions with 1 × 10^6^ CFU/mL *S. aureus*.

### 4.11. Statistical Analysis

Data analysis was performed with Graphpad Prism 6 (GraphPad Software, Inc., La Jolla, CA, USA), and statistical significance was calculated with t test by comparison with the untreated control (* *p* < 0.05; ** *p* < 0.01; *** *p* < 0.001). All experiments were conducted in triplicate.

## Figures and Tables

**Table 1:** Sequences and physicochemical properties of GHa and its analogues.

Peptide	Sequence	MW ^b^	μH ^a^	Amphip-Athicity ^a^	Charge ^a^	PI ^a^	BI ^b^	GRAVY ^b^
GHa	FLQHIIGALGHLF	1464.76	1.71	0.32	1	7.67	−1.49	1.315
GHaK	FLQKIIGALGKLF	1446.83	1.78	0.66	2	10.7	−1.35	1.208
GHa4K	FLQKIIGALGHLF	1455.79	1.74	0.49	1.5	9.87	−1.42	1.262
GHa11K	FLQHIIGALGKLF	1455.79	1.76	0.49	1.5	9.87	−1.42	1.262

^a^ Determined at DBAASP, PI was isoelectric point; ^b^ Determined at APD3, BI is the Boman index (kcal/mol).

**Table 2:** MICs and MBCs of GHa and its analogs against the tested strains.

Species	Strains		(μM) (μg/mL)				
			GHa	GHaK	GHa4K	GHa11K	Kanamycin
Gram+	SA	MIC	12.5 (18.3)	1.6 (2.3)	3.1 (4.5)	1.6 (2.3)	6.2 (3.6)
		MBC	25 (36.6)	1.6 (2.3)	3.1 (4.5)	1.6 (2.3)	
	SM	MIC	25 (36.6)	3.1 (4.5)	6.2 (9.1)	6.2 (9.1)	6.2 (3.6)
		MBC	50 (73.2)	6.2 (9)	6.2 (9.1)	6.2 (9.1)	
	BS	MIC	>100 (>146.5)	12.5 (18.1)	>100 (>145.6)	25 (36.4)	>100 (>58.2)
		MBC	>100 (>146.5)	25 (36.2)	>100 (>145.6)	50 (72.8)	
	MRSA	MIC	100 (146.5)	6.2 (9)	6.2 (9.1)	6.2 (9.1)	>100 (>58.2)
		MBC	>100 (>146.5)	6.2 (9)	6.2 (9.1)	6.2 (9.1)	
	MRSA-2	MIC	>100 (>146.5)	6.2 (9)	12.5 (18.2)	6.2 (9.1)	>100 (>58.2)
		MBC	>100 (>146.5)	12.5 (18.1)	12.5 (18.2)	12.5 (18.2)	
Gram-	EC	MIC	25 (36.6)	6.2 (9)	12.5 (18.2)	12.5 (18.2)	12.5 (7.3)
		MBC	50 (73.2)	6.2 (9)	12.5 (18.2)	12.5 (18.2)	
	D31	MIC	>100 (>146.5)	12.5 (18.1)	>100 (>145.6)	50 (72.8)	25 (14.6)
		MBC	>100 (>146.5)	12.5 (18.1)	>100 (>145.6)	50 (72.8)	
	PAO1	MIC	>100 (>146.5)	12.5 (18.1)	>100 (>145.6)	>100 (>145.6)	25 (14.6)
		MBC	>100 (>146.5)	50 (72.3)	>100 (>145.6)	>100 (>145.6)	
	PA	MIC	>100 (>146.5)	6.2 (9)	6.2 (9.1)	50 (72.8)	>100 (>58.2)
		MBC	>100 (>146.5)	12.5 (18.1)	6.2 (9.1)	>100 (>145.6)	
Fungi	CA	MIC	50 (73.2)	25 (36.2)	>100 (>145.6)	50 (72.8)	>100 (>58.2)
		MBC	>100 (>146.5)	50 (72.3)	>100 (>145.6)	>100 (>145.6)	

SA: *Staphylococcus aureus* (ATCC 25923), SM: *Streptococcus mutans* (ATCC 25175), BS: *Bacillus subtilis* (ATCC 6633), MRSA: methicillin-resistant *Staphylococcus aureus* (ATCC 43300)*,* MRSA-2: methicillin-resistant *Staphylococcus aureus*-2 (clinically isolated), EC: *Escherichia coli* (ATCC 25922), D31: *Escherichia coli* (D31) is an anti-streptomycin strain, PAO1: *Pseudomonas aeruginosa PAO1* (wild-type), PA: *Pseudomonas aeruginosa* (ATCC 15442), CA: *Candida albicans* (ATCC 10231).

**Table 3:** The antibiofilm activity of peptides against *S. aureus*.

Peptide	(μM) (μg/mL)			
	MBIC_50_	MBIC_90_	MBEC_50_	MBEC_90_
GHa	6.2 (9.2)	12.5 (18.3)	50 (73.2)	100 (146.5)
GHaK	0.4 (0.6)	1.6 (2.3)	3.1 (4.5)	12.5 (18.1)
GHa4K	0.8 (1.1)	3.1 (4.5)	12.5 (18.2)	25 (36.4)
GHa11K	0.8 (1.1)	1.6 (2.3)	3.1 (4.5)	12.5 (18.2)

MBIC_50_, minimum biofilm inhibitory concentration required to inhibit the formation of 50% of biofilm; MBIC_90_, minimum biofilm inhibitory concentration required to inhibit the formation of 90% of biofilm; MBEC_50_, minimum biofilm eradication concentration required to eradicate 50% of the formed biofilm; and MBEC_90_, minimum biofilm eradication concentration required to eradicate 90% of the formed biofilm.

**Table 4:** Hemolytic activity of GHa and derived peptides.

		MHC ^a^ (μM) (μg/mL)	HL_50_^b^ (μM) (μg/mL)	CSI ^c^	TI ^d^
GHa	no bacterial	20(29.3)	115(168.4)	9.2	0.5
	with *S. aureus*	29(42.5)	125(183.1)		
GHaK	no bacterial	16(23.1)	66(95.5)	42.3	2.8
	with *S. aureus*	22(31.8)	105(151.9)		
GHa4K	no bacterial	26(37.8)	69(100.4)	22.3	3.7
	with *S. aureus*	70(101.9)	156(227.1)		
GHa11K	no bacterial	15(21.8)	40(58.2)	25.6	2.3
	with *S. aureus*	65(94.6)	149(216.9)		

^a^ The minimum hemolytic concentration that caused 10% hemolysis of hRBC; ^b^ The concentration at which 50% of hRBCs were lysed; ^c^ CSI: calculated using the ratio of HL_50_ to the MIC of the peptide against *S. aureus* (ATCC 25923); ^d^ The ratio of the MHC to the geometric mean of the MICs (GM).

## Supplementary Materials

The following are available online at https://www.mdpi.com/1420-3049/24/22/4173/s1, The following are available online at https://www.mdpi.com/1420-3049/24/22/4173/s1, Figure S1. Bioinformatics statistics of antimicrobial peptide properties, Figure S2. Amino acid profile analysis of the AMPs of small data set and sequence logo of the temporin family AMPs, Figure S3. The concentration of the bacteria was measured at 600 nm while the membrane permeability was detected, Figure S4. The RP-HPLC chromatogram (**A**) and mass spectrum (**B**) of Gha, Figure S5. The RP-HPLC chromatogram (**A**) and mass spectrum (**B**) of GhaK, Figure S6. The RP-HPLC chromatogram (**A**) and mass spectrum (**B**) of GHa4K, Figure S7. The RP-HPLC chromatogram (**A**) and mass spectrum (**B**) of GHa11K, Table S1. Antimicrobial activity prediction by CAMP_R3_, Table S2. Antimicrobial activity prediction by DBAASP and antibiofilm activity prediction by dPABBs, Table S3. Growth curve analysis.
