# Supplementary material for: The Analogs of Temporin-GHa Exhibit a Broader Spectrum of Antimicrobial Activity and a Stronger Antibiofilm Potential against Staphylococcus aureus
Source: Molecules. 2019 Nov 18;24(22):4173. doi: 10.3390/molecules24224173 (PMC6891419; doi:10.3390/molecules24224173)
Supplement: Supplementary file 1 [file molecules-24-04173-s001.pdf]

molecules

The Analogs of Temporin-GHa Exhibit a Broader Spectrum of Antimicrobial Activity and a Stronger Antibiofilm Potential against *Staphylococcus aureus*

Zhipeng Xie [1], Hanqi Wei [1], Jiahui Meng [1], Tong Cheng [1], Yanting Song [1], Manchuriga Wang [2,*] and Yingxia Zhang [1,*]

[1] Key Laboratory of Tropical Biological Resources of Ministry of Education; School of Life and Pharmaceutical Sciences, Hainan University, Haikou 570228, China; xiezhipeng1994@163.com (Z.X.), w370694686@163.com (H.W.), frank1026855189@gmail.com (J.M.), ct1498156623@163.com (T.C.), songyanting3323@hotmail.com (Y.S.)

[2] College of Animal Science and Technology, Hainan University, Haikou 570228, China

* Correspondence: manchuriga@163.com (M.W.); zhangyingxia@hainanu.edu.cn (Y.Z.)

Received: 21 October 2019; Accepted: 15 November 2019; Published: 18 November 2019

TABLE OF CONTENTS:

Figure S1: Bioinformatics statistics of antimicrobial peptide properties 2
Figure S2: Amino acid profile analysis of the AMPs of small data set and sequence logo of the temporin family AMPs 2
Table S1: Antimicrobial activity prediction by CAMP$_{R3}$ 2
Table S2: Antimicrobial activity prediction by DBAASP and antibiofilm activity prediction by dPABBs. 3
Table S3: Growth curve analysis. 3
Figure S3: The concentration of the bacteria was measured at 600 nm while the membrane permeability was detected. 3
Figure S4: The RP-HPLC chromatogram (A) and mass spectrum (B) of GHa 4
Figure S5: The RP-HPLC chromatogram (A) and mass spectrum (B) of GHaK 5
Figure S6: The RP-HPLC chromatogram (A) and mass spectrum (B) of GHa4K 6
Figure S7: The RP-HPLC chromatogram (A) and mass spectrum (B) of GHa11K 7

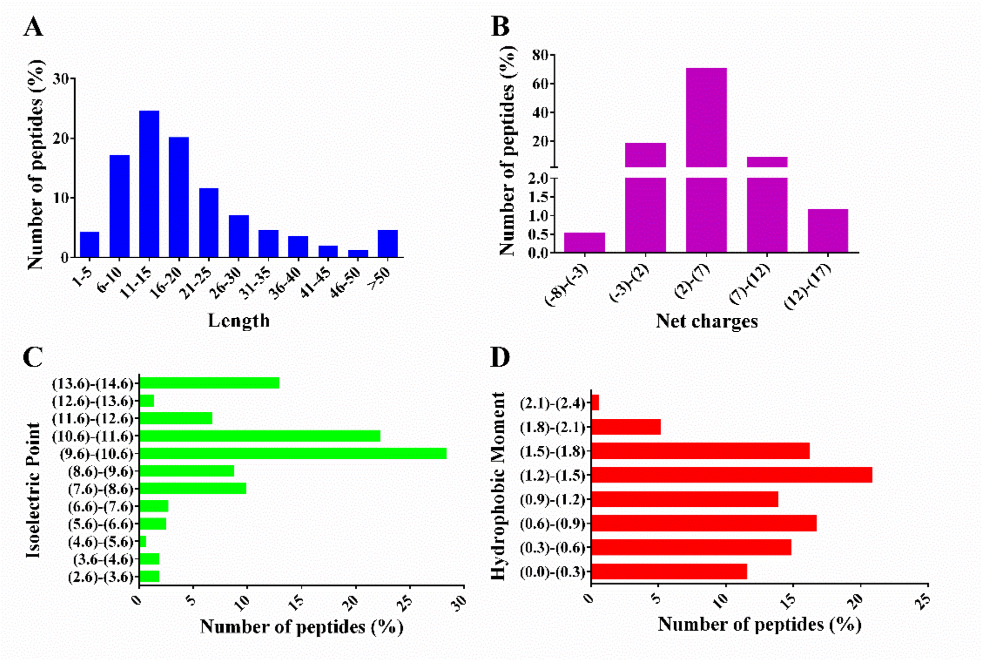

Figure S1. Bioinformatics statistics of antimicrobial peptide properties were represented by (**A**) length, (**B**) net charge, (**C**) isoelectric point, and (**D**) hydrophobic moment. Visited on January 19, 2019.

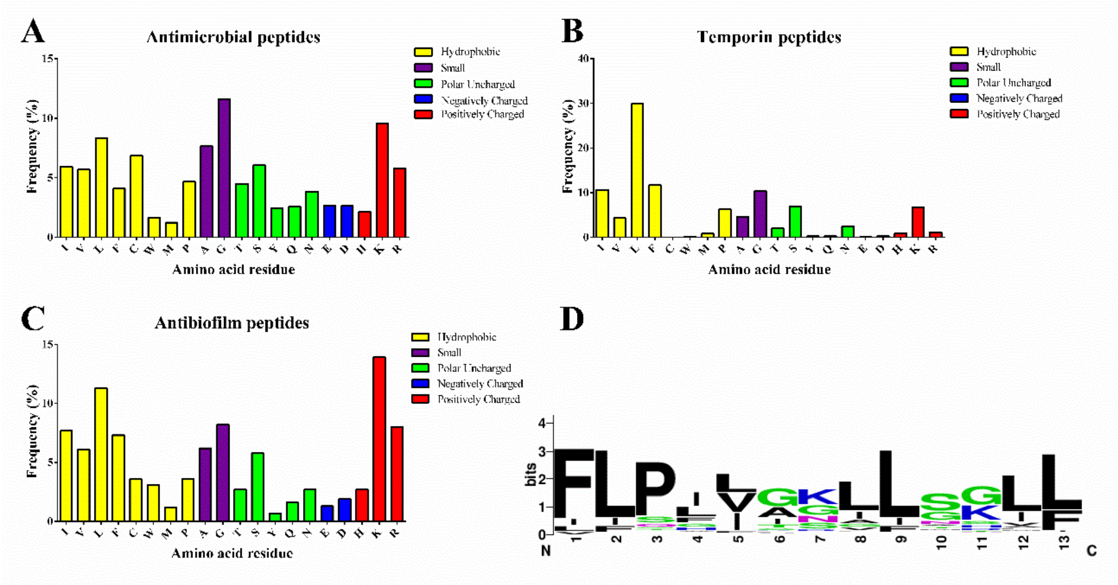

Figure S2. Amino acid profile analysis of the AMPs of small data set and sequence logo of the temporin family AMPs. (**A**) The amino acid spectrum of AMPs, (**B**) temporin family, and (**C**) the antibiofilm peptides were analyzed by using APD3. (**D**) The sequence logo of 122 temporin family AMPs was analyzed by WebLogo. Visited on January 19, 2019.

Table S1. Antimicrobial activity prediction by CAMP$_{R3}$.

Peptides	SVM	RF	ANN	DA
GHa	0.927	0.7685	AMP	0.971
GHaK	0.968	0.971	AMP	0.995
GHa4K	0.944	0.944	AMP	0.995
GHa11K	0.966	0.987	AMP	0.998

Higher probability scores (between 0 and 1) mean that the peptide is more likely to exert antimicrobial activity.

Table S2. Antimicrobial activity prediction by DBAASP and antibiofilm activity prediction by dPABBs.

Peptides	Antimicrobial Activity				Antibiofilm Activity	
	S. aureus	*B. Subtilis*	*E. coli*	*P. aeruginosa*	SVM Score	WEKA-Based
GHa	+	−	−	−	1.01 (+)	0.37 (−)
GHaK	+	+	+	+	1.15 (+)	0.75 (+)
GHa4K	+	+	-	−	1.23 (+)	0.71 (+)
GHa11K	+	+	+	−	1.23 (+)	0.71 (+)

+ means active, − means inactive. dPABBs: http://ab-openlab.csir.res.in/abp/antibiofilm/index.php. SVM means support vector machine, which was carried out by the means of the SVM version 6.02 software. Weka is a Java based software package with various machine learning and feature selection algorithms for data mining tasks. Higher probability scores in SVM score and WEKA-based mean that the peptide is more likely to exert antibiofilm activity.

Table S3. Growth curve analysis.

Peptide	Time to Reach the Stationary Phase (h)			The Lag Phase Length (h)		
	1/2 MIC	1/4 MIC	1/8 MIC	1/2 MIC	1/4 MIC	1/8 MIC
GHa	22	20	20	12	10	10
GHaK	24	24	20	17	13	11
GHa4K	23	23	20	15	12	11
GHa11K	24	24	20	17	15	11
Control		20			10	

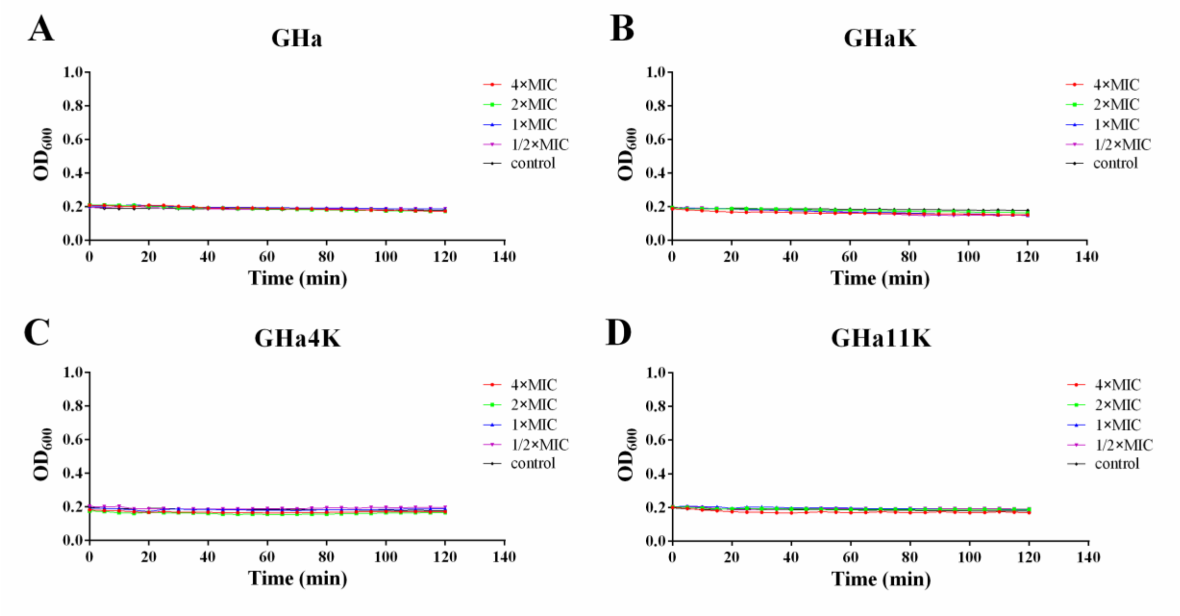

Figure S3. The concentration of the bacteria was measured at 600 nm while the membrane permeability was detected. Bacteria were treated by (**A**) GHa, (**B**) GHaK, (**C**) GHa4K, and (**D**) GHa11K at various concentrations.

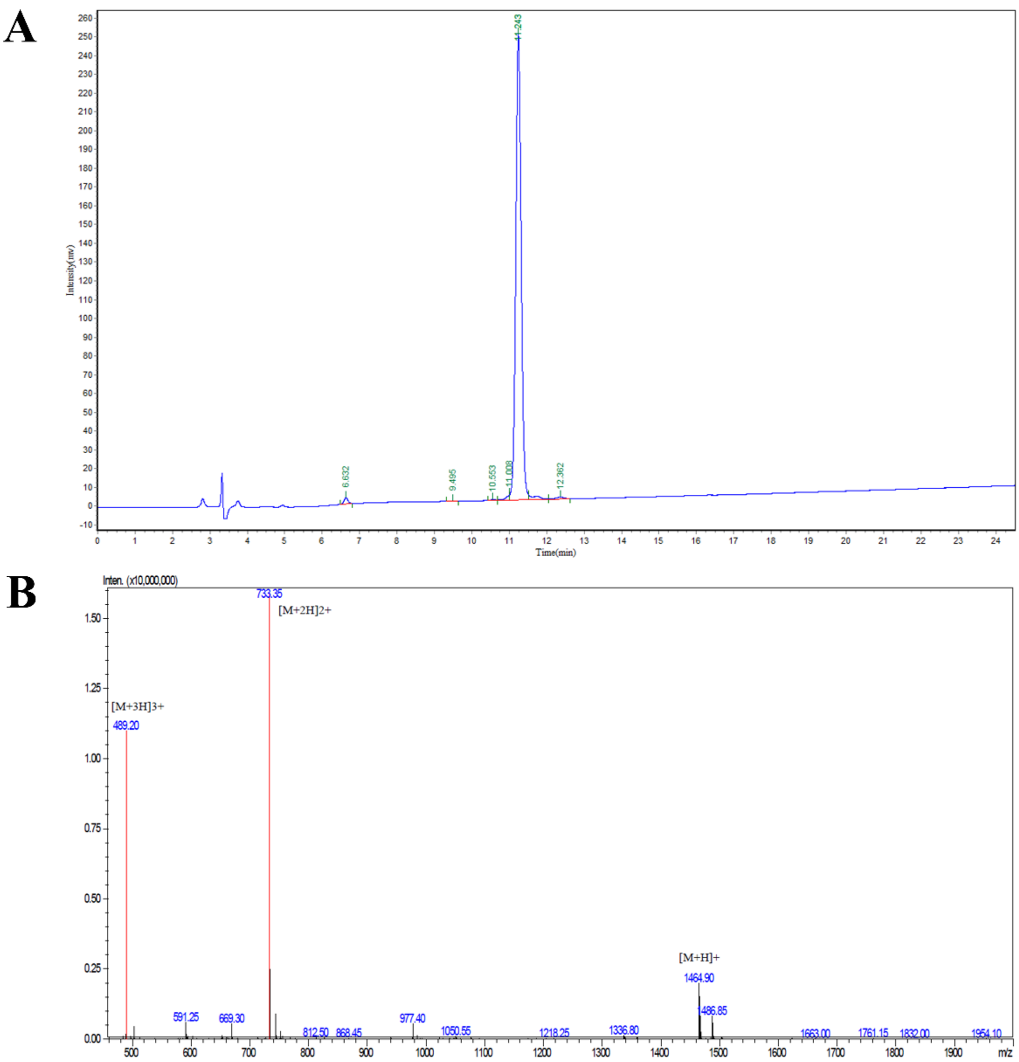

Figure S4. The RP-HPLC chromatogram (**A**) and mass spectrum (**B**) of GHa. Mw is 1464.75 Da. Peptide purity is 96.20%.

A

B

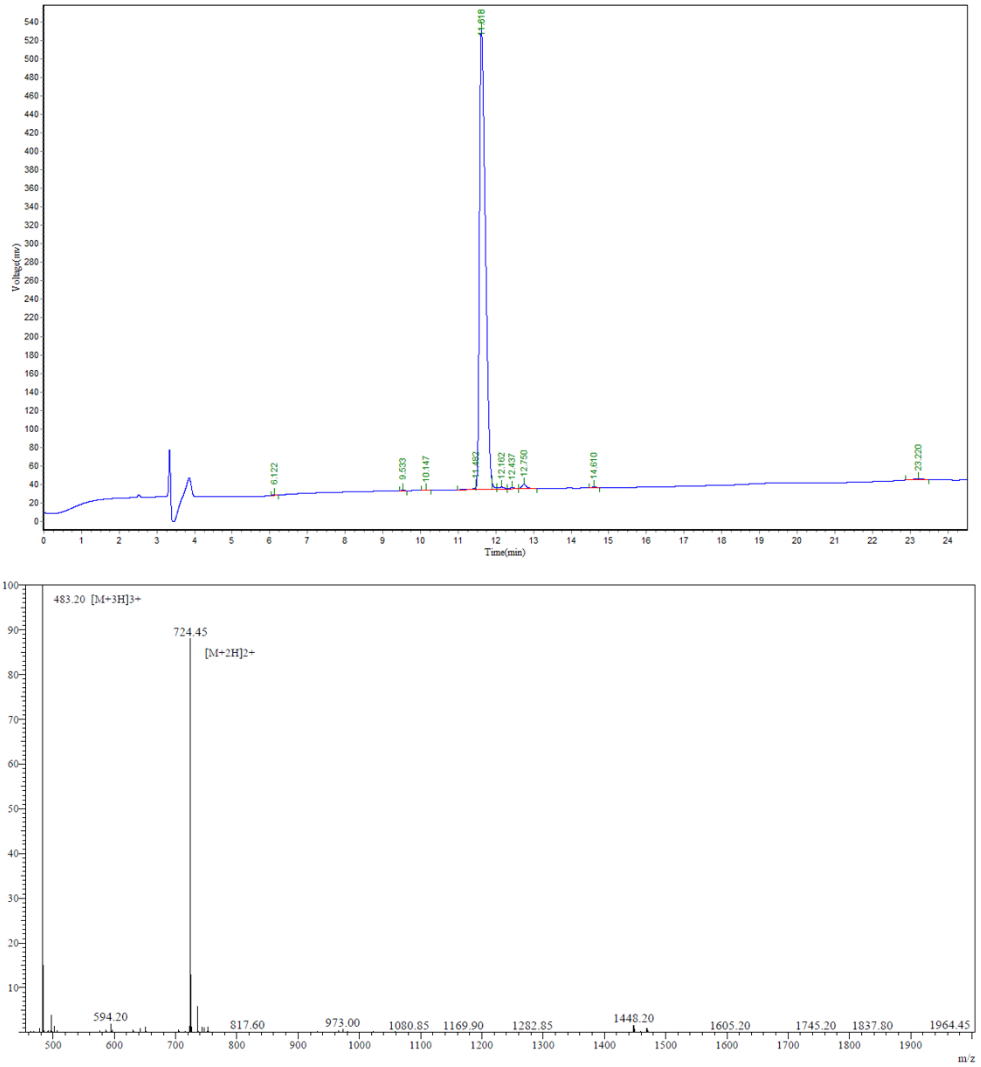

Figure S5. The RP-HPLC chromatogram (**A**) and mass spectrum (**B**) of GHaK. Mw is 1446.86 Da. Peptide purity is 97.29%.

A

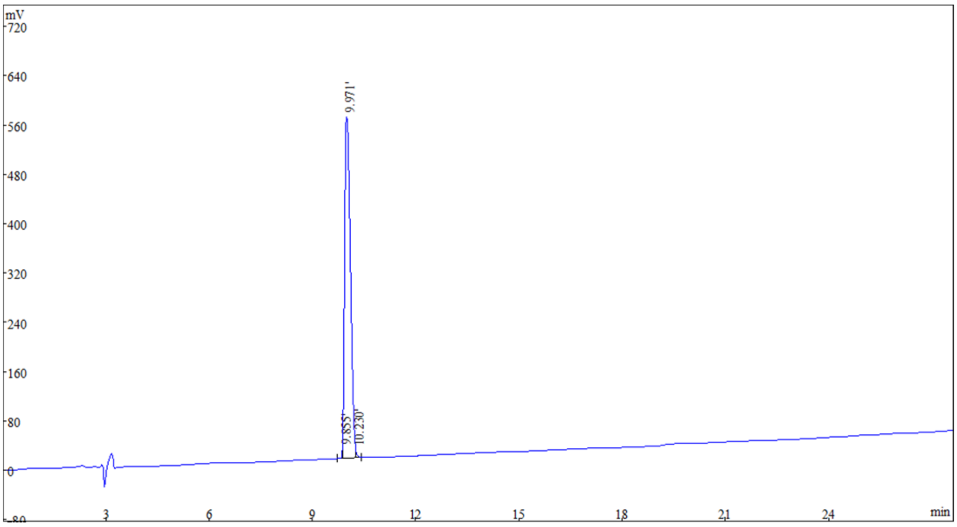

B

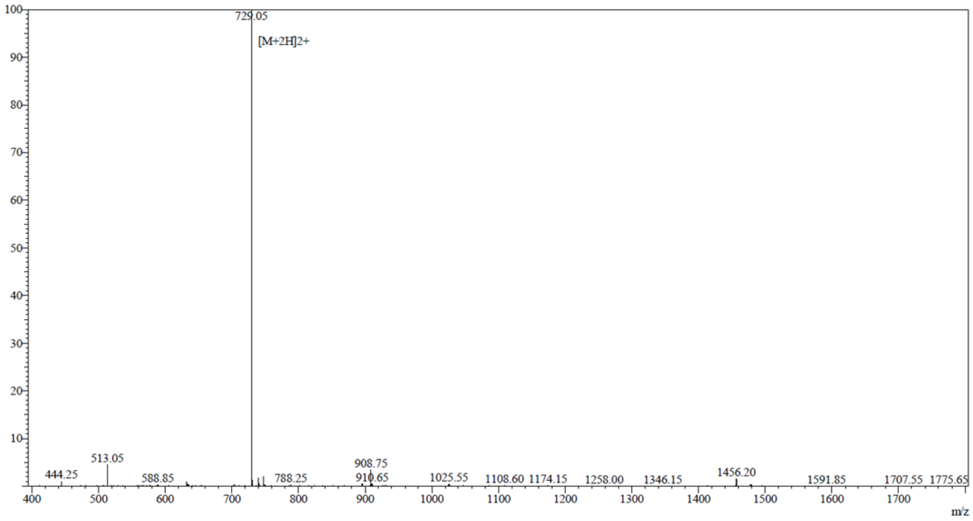

Figure S6. The RP-HPLC chromatogram (**A**) and mass spectrum (**B**) of GHa4K. Mw is 1455.79 Da. Peptide purity is 99.40%.

A

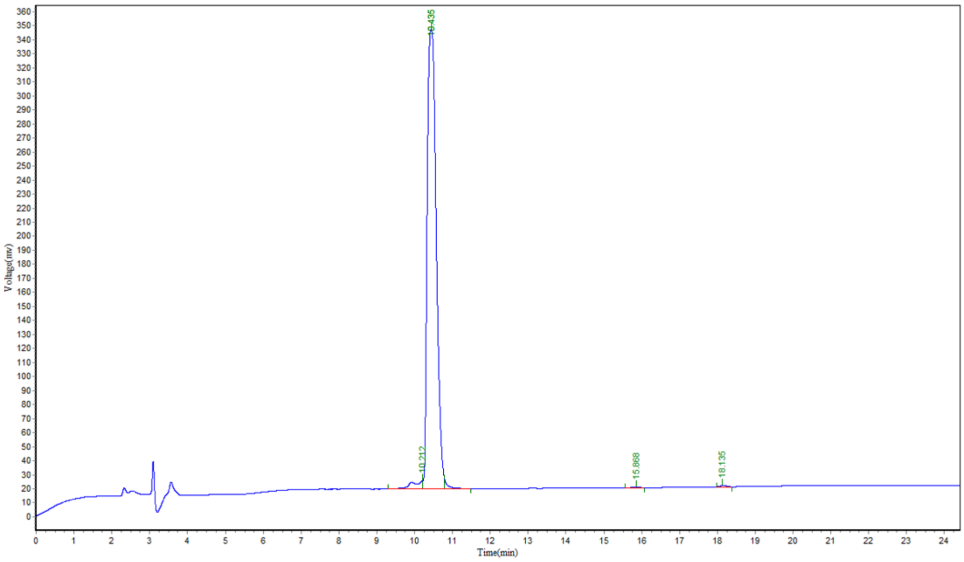

B

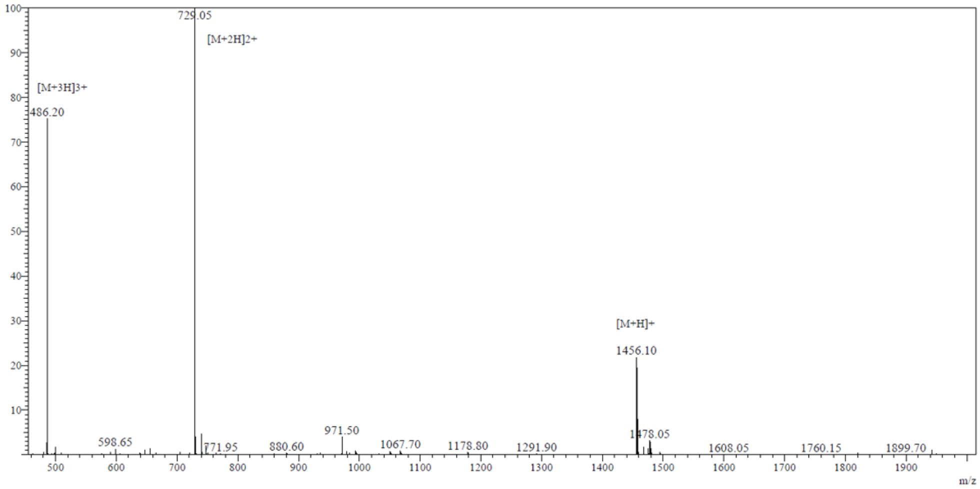

Figure S7. The RP-HPLC chromatogram (**A**) and mass spectrum (**B**) of GHa11K. Mw is 1455.82 Da. Peptide purity is 96.92%.

© 2019 by the authors. Submitted for possible open access publication under the terms and conditions of the Creative Commons Attribution (CC BY) license (http://creativecommons.org/licenses/by/4.0/).
